# Contact-dependent carcinoma aggregate dispersion by M2a macrophages via ICAM-1 and β2 integrin interactions

**DOI:** 10.18632/oncotarget.4716

**Published:** 2015-07-30

**Authors:** Jing Bai, Giulia Adriani, Truong-Minh Dang, Ting-Yuan Tu, Hwei-Xian Leong Penny, Siew-Cheng Wong, Roger D. Kamm, Jean-Paul Thiery

**Affiliations:** ^1^ BioSystems and Micromechanics IRG, Singapore-MIT Alliance for Research and Technology, 138602, Singapore; ^2^ Department of Biological Engineering, Massachusetts Institute of Technology, Cambridge, MA, 02139, USA; ^3^ SIgN (Singapore Immunology Network), A*STAR (Agency for Science, Technology and Research), Biopolis, 138648, Singapore; ^4^ Department of Biochemistry, Yong Loo Lin School of Medicine, National University of Singapore, 117597, Singapore; ^5^ Institute of Molecular and Cell Biology, Proteos, 138673, Singapore

**Keywords:** epithelial-mesenchymal transition, microfluidics, macrophage phenotypes, macrophage polarization, cancer microenvironment

## Abstract

Tumor-associated macrophages (TAMs) can constitute up to 50% of the tumor mass and have strong implications in tumor progression and metastasis. Macrophages are plastic and can polarize to various subtypes that differ in terms of surface receptor expression as well as cytokine and chemokine production and effector function. Conventionally, macrophages are grouped into two major subtypes: the classically activated M1 macrophages and the alternatively activated M2 macrophages. M1 macrophages are pro-inflammatory, promote T helper (Th) 1 responses, and show tumoricidal activity, whereas M2 macrophages contribute to tissue repair and promote Th2 responses. Herein, we present a microfluidic system integrating tumor cell aggregates and subtypes of human monocyte-derived macrophages in a three-dimensional hydrogel scaffold, in close co-culture with an endothelial monolayer to create an *in vitro* tumor microenvironment. This platform was utilized to study the role of individual subtypes of macrophages (M0, M1, M2a, M2b and M2c) in human lung adenocarcinoma (A549) aggregate dispersion, as a representation of epithelial-mesenchymal transition (EMT). A significant difference was observed when M2a macrophages were in direct contact with or separated from A549 aggregates, suggesting a possible mechanism for proximity-induced, contact-dependent dissemination via ICAM-1 and integrin β2 interactions. Indeed, M2a macrophages tended to infiltrate and release cells from carcinoma cell aggregates. These findings may help in the development of immunotherapies based on enhancing the tumor-suppressive properties of TAMs.

## INTRODUCTION

Epithelial-mesenchymal transition (EMT) plays a critical role in the early stages of tumor dissemination [1]. During EMT, carcinoma cells lose their cell-cell junctions and acquire an invasive fibroblast-like morphology in the adjacent stroma. Subsequently, carcinoma cells may intravasate into blood and lymph vessels and disseminate to distant organs. Carcinoma cells undergoing EMT will acquire clonogenic and stem cell-like properties, escape immune surveillance and become refractory to treatment [2]. EMT is generally promoted via complex signaling networks involving tyrosine kinase receptors, growth factors and extracellular matrix (ECM) components [3, 4]. Interestingly, it has been shown that tumor-associated macrophages (TAMs), which constitute the major component of infiltrated cells, contribute to EMT [5–7]. In fact, a tumor microenvironment of metastasis was observed to occur where there is a tripartite arrangement of an invasive cancer cell, a macrophage and an endothelial cell [8, 9]. Despite recent studies on TAMs and other bone marrow-derived cells that promote EMT [6, 10, 11] and cancer metastasis [12–16], the regulatory mechanisms controlling TAM activation in response to a malignant environment have yet to be fully defined, as has their role in cancer.

In certain cases, TAMs exhibit an M1 phenotype, are pro-inflammatory, and have an anti-tumoral role, as shown *in vitro* for colon cancer [17, 18] and in patients with colorectal and gastric cancers [19, 20]. However, in numerous other cancers, such as breast [21], endometrial [22] and lung [6], macrophages acquire an alternative M2 phenotype that promotes EMT invasion and metastasis, thus leading to a poor prognosis. M2 macrophages are further classified into M2a, M2b, and M2c based on the factors that promoted their polarization [23, 24].

Although significant progress has been made in identifying the chemokine repertoire that generates the diverse types of macrophages [25], their specific mechanisms of action in carcinoma cell dissemination remain unknown.

Here, we employed human lung adenocarcinoma (A549) cell aggregates to assess the role of distinct TAMs in inducing EMT and carcinoma cell dissemination. A three-dimensional (3D) microfluidic platform was created, integrating carcinoma cell aggregates, macrophages and human umbilical vein endothelial cells (HUVECs). Aside from enabling the dynamic visualization of carcinoma cell aggregate dispersion and the interaction between carcinoma cells and macrophages, this system provided significant advantages over other platforms with the opportunity for real-time monitoring and precise measurements of cell to cell distances [26]. Using this system, we found that M1 and M2b macrophages promoted the greatest dispersal of A549 carcinoma aggregates, regardless of their proximity to the aggregates. More interestingly, M2a macrophages promoted significant A549 carcinoma aggregate dispersal only when they were in contact with the carcinoma aggregates, and required integrins for allowing contact-dependent dissemination. Our results provide the first step towards a better understanding of allowing pathogenic roles of different macrophage subtypes and may aid in the design of novel cancer therapeutic treatments.

## RESULTS

### Stability of the 3D microfluidic-based tumor microenvironment

**Figure 1:**
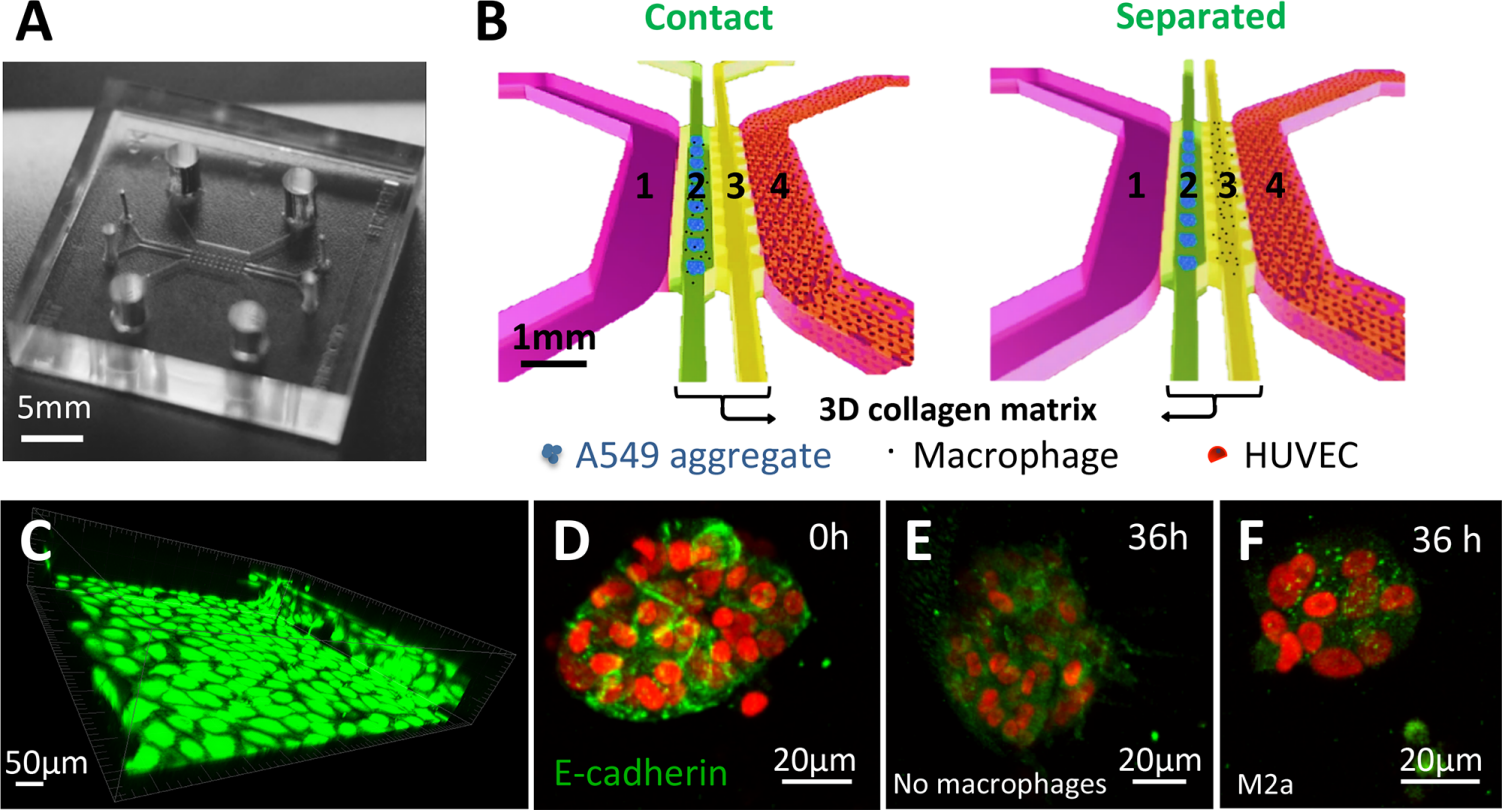
Microfluidic co-culture platform to study the interactions between carcinoma aggregates and macrophages **A.** Photograph of the polydimethyl siloxane (PDMS) device. **B.** Schematic images of an enlarged, isometric view of the channel layout showing the orientation of co-culturing carcinoma cell aggregates and endothelial cells (HUVECs), with macrophages either physically contacting (left panel; 1: media channel; 2: A549 aggregates and macrophages gel channel; 3. supporting gel channel; 4: HUVEC monolayer) or cultured under separated conditions (right panel; 1: media channel; 2: A549 aggregates gel channel; 3. macrophages gel channel; 4: HUVEC monolayer). **C.** HUVEC monolayers formed in the microfluidic channel. Green: GFP-HUVECs. **D–F.** E-cadherin immunocytochemical staining. E-cadherin expression of A549 aggregates at 0 h (D), E-cadherin expression of A549 aggregates in the absence (E) or presence (F) of macrophages at 36 h. Green: E-cadherin staining, red: mCherry A549 nuclei.

To investigate whether macrophages contribute to cancer cell EMT, we selected A549 lung carcinoma cells, as they exhibit a reversible EMT phenotype. In addition, TAMs have also been shown to promote EMT in non-small cell lung cancer [6]. For this purpose, a microfluidic platform was designed with two parallel adjacent compartments containing 3D collagen matrices, flanked by channels for culture media (Figure 1A, 1B). In a previous study using a similar system [26], we demonstrated that HUVECs could promote carcinoma aggregate dispersal using a single collagen compartment. Here, we undertook a more integrative approach, where HUVECs were cultured in one of the media channels (Figure 1B, 1C) to mimic cancer cell proximity to a blood capillary, and we injected macrophages and A549 carcinoma aggregates into the collagen compartments. Human primary monocyte-derived macrophages (M0) were further polarized into either M1 or M2 (M2a, M2b, M2c) phenotypes before being introduced into the collagen compartment [24]. Two experimental configurations were set-up: (i) a “contact condition”, where macrophages and carcinoma aggregates were introduced and co-cultured within a single collagen compartment, (Figure 1B, left panel) and (ii) a “separated condition”, where the two cell types were placed in two separate but adjacent collagen compartments, with macrophages in the compartment next to the channel containing the HUVECs (Figure 1B, right panel). Such topographical arrangement partially reconstitutes an *in vivo* tumor microenvironment and thus provides an opportunity to examine the nature of macrophage/carcinoma cell interactions. Live and dead cell assays revealed good viability of M0 cells within the microfluidic device, even up to 36 h in culture (Supplementary Figure S2).

**Figure 2:**
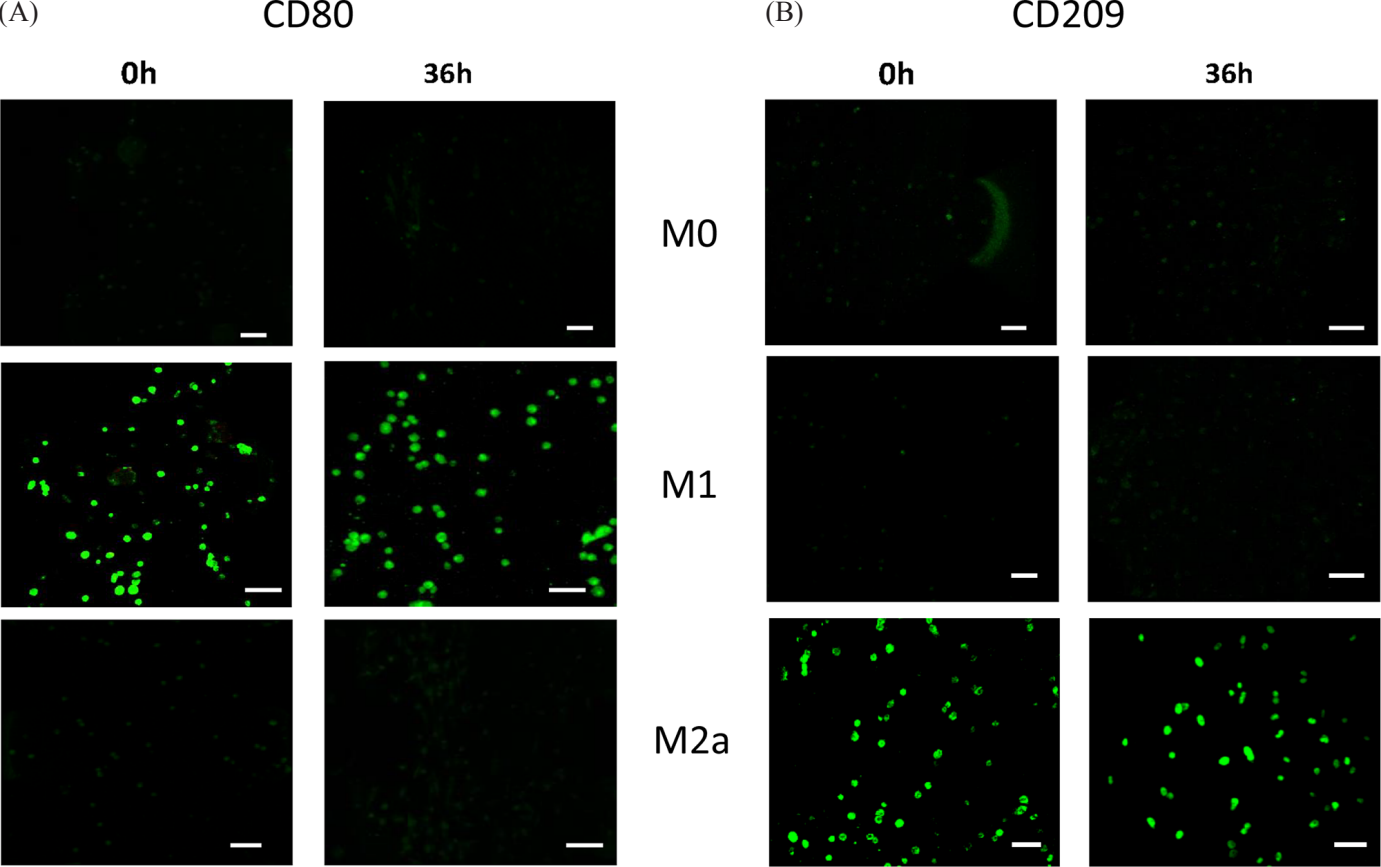
Characterization of polarized macrophages M0, M1 and M2a macrophages were immunostained for markers at either 0 h or after culturing for 36 h in the microfluidic device in co-culture with A549 carcinoma aggregates: **A.** CD80 (M1 marker); **B.** CD209 (M2a marker). Scale bars 50 μm.

Consistently, immunostaining using CD80, a marker specific for M1, and CD209, a marker specific for M2a, showed that the various macrophages retained the expression of their respective markers after 36 h, even within the 3D collagen matrix in the presence of carcinoma aggregates and HUVECs (Figure 2).

### Polarized macrophages differentially induce A549 carcinoma aggregate dispersal

**Figure 3:**
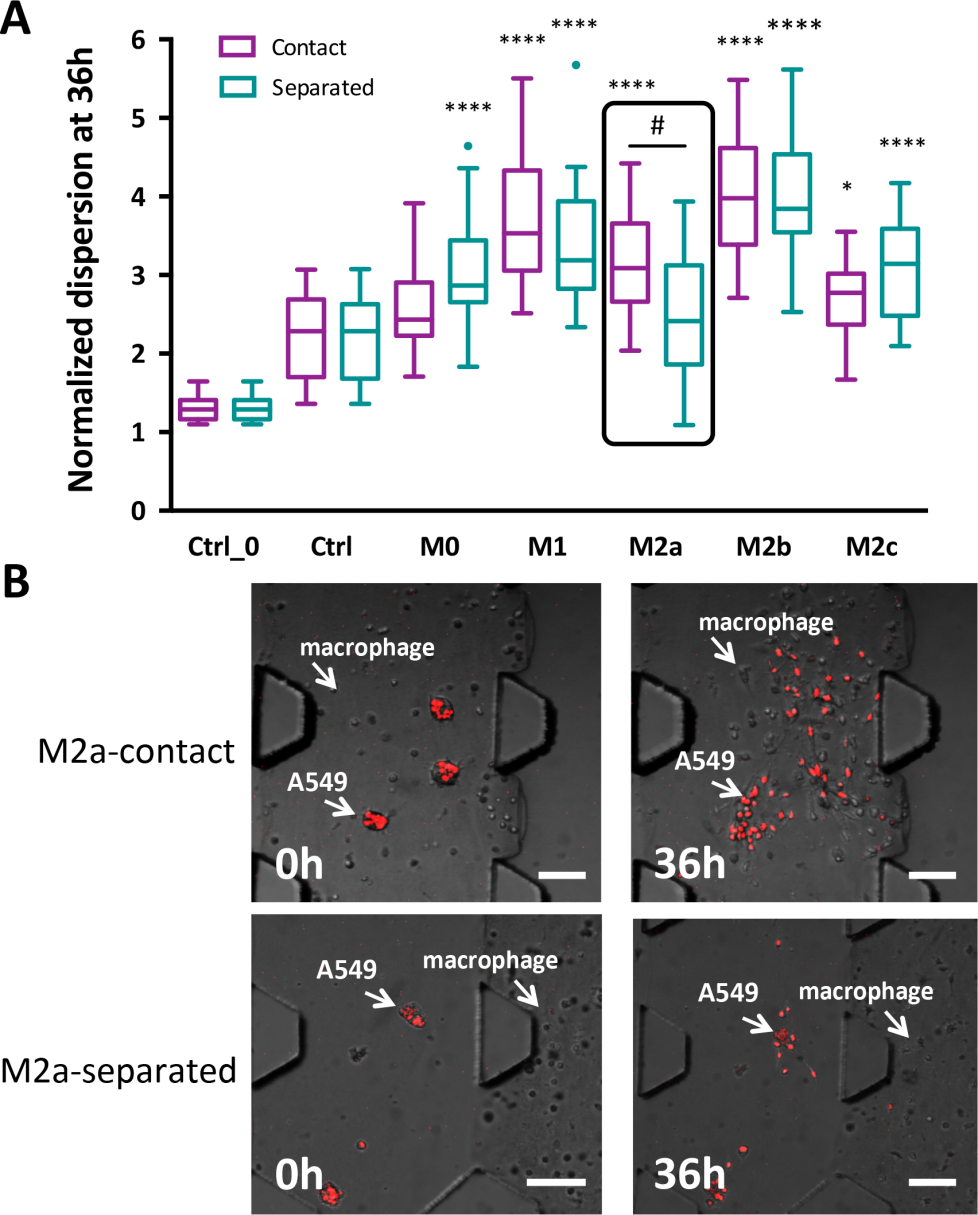
A549 aggregates dispersion induced by various subtypes of macrophages **A.** Quantitative measurement of aggregate dispersion at 36 h for macrophages under either “contact” or “separated” conditions. * indicates statistical calculations compared to no macrophage conditions, where *P < 0.05 and ****P < 0.0001. # indicates a statistical calculation between “contact” versus “separated” culture conditions, where #*P* < 0.01. Data are shown as a box plot with Tukey outliers. Ctrl_0 represents the control without HUVECs and without macrophages. Ctrl represents the control with HUVECs but without macrophages. **B.** Images of M2a inducing A549 aggregate dispersion under “contact” and “separated” conditions at 0 h or after culture for 36 h. Red: mCherry A549 nuclei. Scale bars 100 μm.

To determine the effect of macrophages on A549 carcinoma aggregate dispersal, the two cell types were cultured under “contact” or “separated” conditions, as described above. Figure 3A summarizes the normalized dispersion of aggregates when macrophages were grown under these conditions. Similar to what we had previously described, A549 aggregates exhibited greater dispersion in the presence of HUVECs (Ctrl in Figure 3A) versus the absence of HUVECs (Ctrl_0 in Figure 3A). When different subtypes of macrophages were included in the device with A549 cells and HUVECs, we found that M1 and M2b macrophage subtypes specifically induced the greatest dispersion under both “contact” and “separated” conditions. Both subtypes induced a normalized dispersal measurement that was three to four times greater than that of the control (no macrophages). Unpolarized macrophages (M0) and M2c macrophages also promoted A549 aggregate dispersion, but the extent of dispersion under both conditions was lower than that observed with the M1 and M2b macrophages. Interestingly, M2a macrophages under “contact” conditions showed increased aggregate dispersal similar to that of M1 macrophages, but the dispersal was significantly lower when M2a cells were grown under “separated” conditions (Figure 3A and 3B). Similar aggregate dispersions were also observed without HUVECs in the 3D culture (Supplementary Figure S4). Representative images of the aggregate dispersion in “contact” conditions for the other macrophage subtypes are in Supplementary Figure S3.

### M2a macrophages preferentially migrate towards carcinoma aggregates

**Figure 4:**
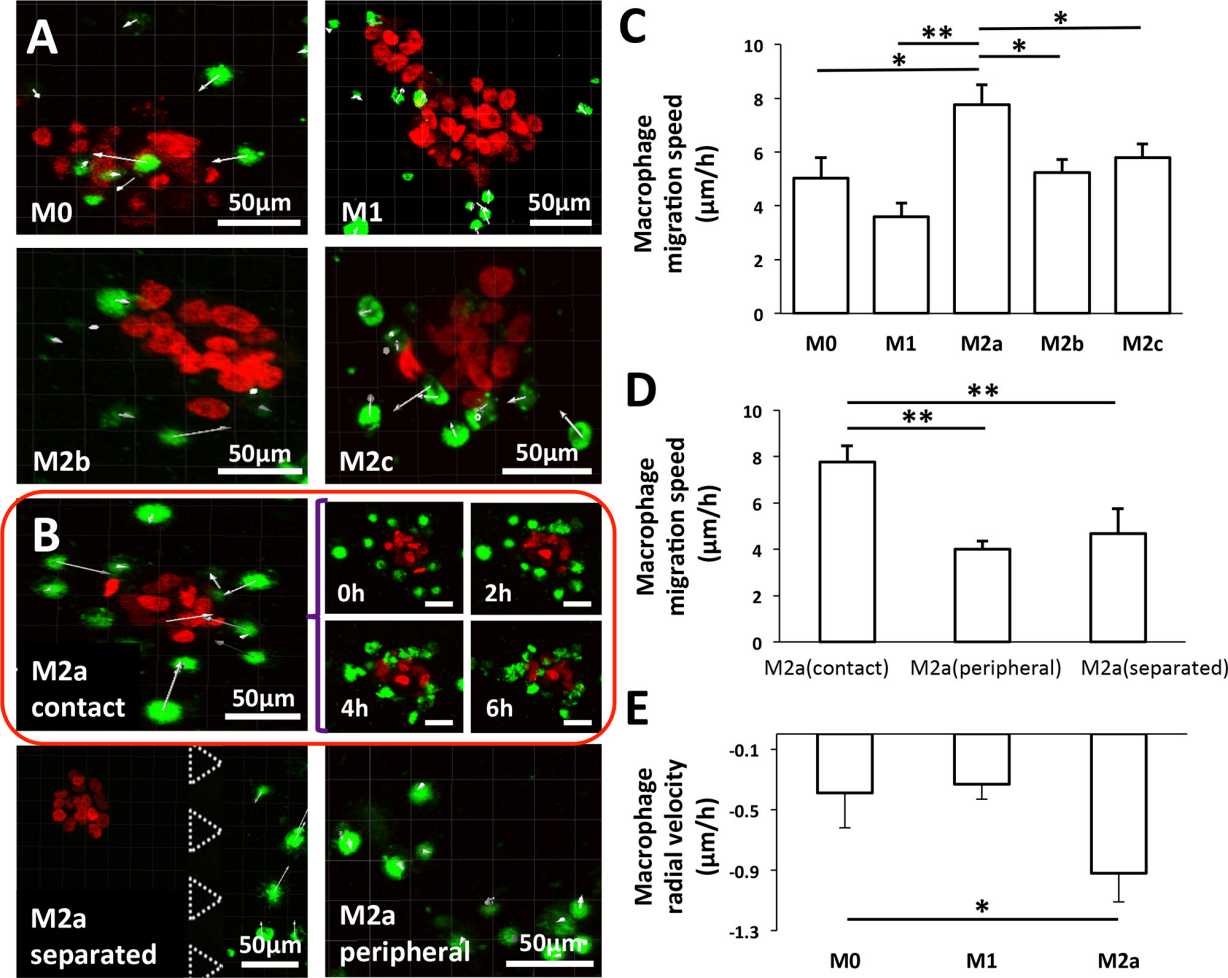
Migration of macrophage subtypes **A.** Migration direction (white arrows) of M0, M1, M2b, and M2c subtypes of macrophages under “contact” conditions with the carcinoma aggregates imaged at 6 h. **B.** Time-lapsed images of the M2a subtype under “contact” condition at specific times (top left). The migration direction (white arrows) of the M2a subtype that were ≤50 μm (M2a-proximity; top right) or ≥50 μm (M2a-peripheral; bottom left) from the carcinoma aggregate under “contact” conditions or M2a cells under “separated” conditions (bottom right). Cells were imaged for 6 h. **C.** Migration speed of macrophages situated ≤50 μm from the carcinoma aggregates. **D.** Migration speed of M2a cells situated either ≤50 μm (M2a-proximity) or ≥50 μm (M2a-peripheral) from the carcinoma aggregates and grown under “separated” conditions. **E.** Radial velocity of M0, M1 and M2a subtypes under “contact” conditions. All data shown are mean ± SEM of at least three experiments. *P < 0.05, **P < 0.01. Green: DiO-labeled macrophages, red: mCherry A549 nuclei.

To capture the 3D dynamics of macrophage migration and carcinoma aggregate dispersion, time-lapse images were taken every 10 min over a 12-h period under “contact” conditions (Supplementary Video S1). M2a macrophages located ≤50 μm from the carcinoma aggregate appeared to be the most motile, with an average migration speed of 7.7 μm/h. M2b and M2c motility varied between 5.2 and 5.8 μm/h, whereas M0 macrophages migrated at 5 μm/h; M1 macrophages exhibited the slowest speed of 3.6 μm/h (Figure 4C and Supplementary Video S1). In contrast, the motility of M2a macrophages located either ≥50 μm from the aggregates in the same compartment or seeded in the compartment adjacent to carcinoma aggregates was significantly lower (4–4.6 μm; Figure 4D). However, a reduction in speed was not observed for the other macrophage subtypes located at a distance ≥50 μm from the carcinoma aggregates (Supplementary Figure S5).

M2a macrophages within close proximity to the carcinoma aggregates were not only more motile, but they also exhibited directional migration towards the A549 aggregates (white arrows, top left, Figure 4B). In contrast, no directional migration was observed for the other macrophage subtypes (Figure 4A, Supplementary Figure S6). To confirm that M2a macrophages indeed exhibited directional migration, we further calculated the radial velocity of the cells (Supplementary Material), with negative values indicating macrophage migration towards the center of the A549 aggregate. When compared to M0 and M1 macrophages, M2a macrophages showed a greater tendency to migrate towards the center of the carcinoma aggregate with an average radial velocity of −0.92 μm/h compared to −0.33 and −0.39 μm/h for M1 and M0 macrophages, respectively (Figure 4E; Supplementary Video S1). Apart from migrating towards the aggregates, M2a macrophages were also observed to establish cell-cell contact with the aggregate. Following contact, A549 cells were found to detach from the aggregate.

### M2a macrophages induce aggregate dispersion via a contact-mediated mechanism

To first assess whether M2a macrophages in contact with A549 aggregate promote EMT, immunocytochemical staining was performed on A549 aggregates in the presence or absence of M2a macrophages at 0 h and 36 h. The loss of expression of the epithelial marker E-cadherin was used to establish the occurrence of EMT (Figure 1D–1F, Green: E-cadherin staining, red: mCherry A549 nuclei). A549 aggregates at 0 h showed clear E-cadherin expression on their surfaces (Figure 1D). E-cadherin expression remained up-regulated over 36 h in the absence of macrophages (Figure 1E). On the other hand, E-cadherin expression in A549 aggregates was down-regulated at 36 h in “contact” conditions with M2a macrophages (Figure 1F), suggesting that the EMT of A549 cancer cells was induced by the presence of M2a macrophages within close proximity.

**Figure 5:**
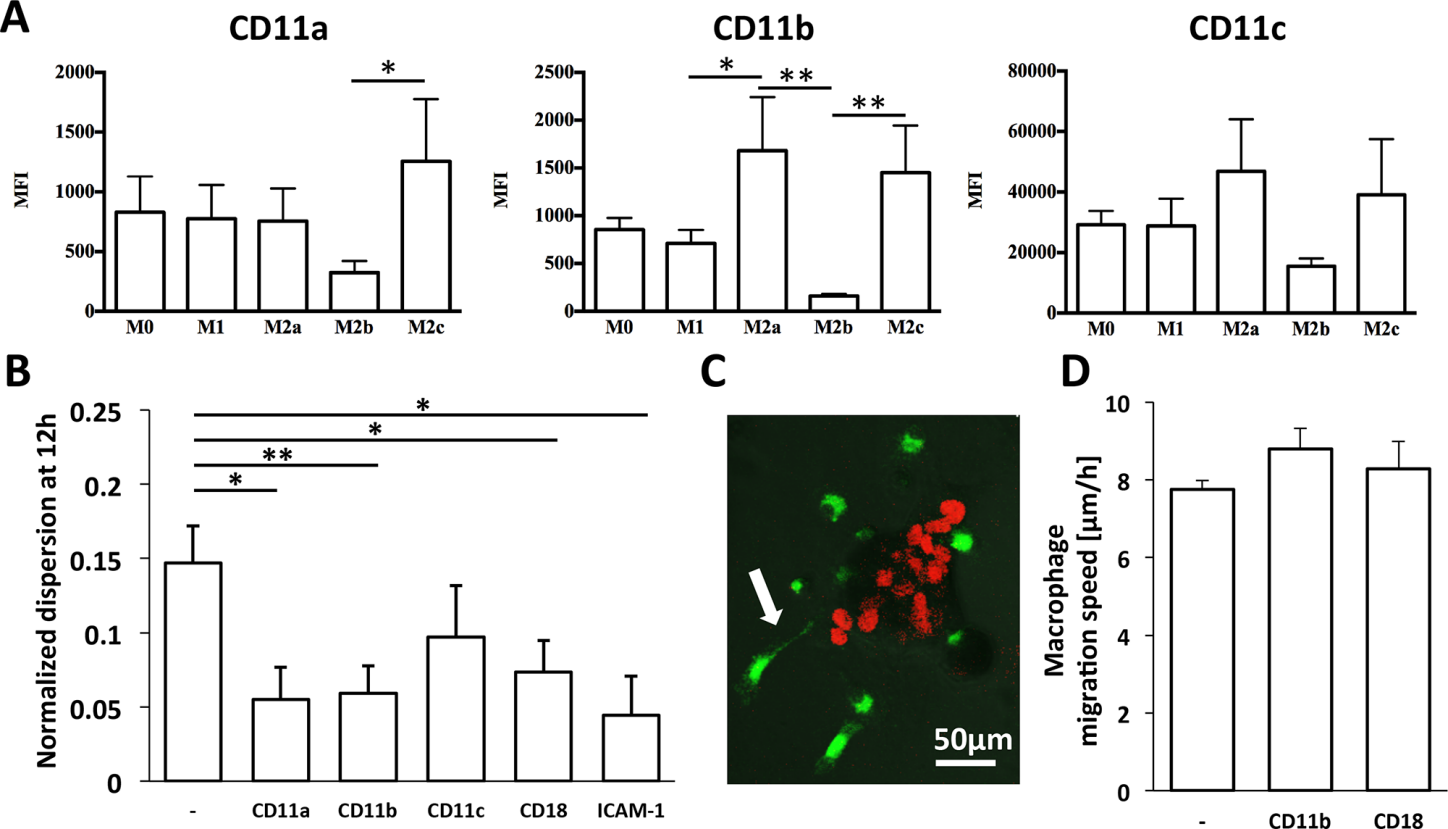
Involvement of integrins in carcinoma aggregate dispersion by macrophages **A.** Surface expression of CD11a, CD11b, and CD11c integrins on macrophage subtypes, as determined by FACS and expressed as mean fluorescence intensity (MFI). **B.** Normalized aggregate dispersion induced by M2a macrophages in the absence (−) or presence of various blocking antibodies at 12 h. *P <0.05 and **P < 0.01. **C.** Fluorescent image showing an M2a macrophage extending a projection (white arrow) as it attempts to dissociate a cancer cell from the aggregate. Green: DiO-labeled macrophages, red: mCherry A549 nuclei. **D.** Migration speed of M2a macrophages in the absence or presence of the indicated blocking antibodies. Data shown are mean ± SEM of three experiments.

To confirm whether aggregate dispersal in the presence of M2a macrophages was indeed dependent on cell-cell contact and to ascertain a role for adhesion in the process, we used flow cytometry to assess the surface expressions of CD11a, CD11b and CD11c integrins on macrophages; these proteins are expressed on leukocytes and interact with ICAM-1 on tumor cells (Figure 5A). We found that CD11a expression was highest on M2c macrophages, whereas CD11b and CD11c were highly expressed on both M2a and M2c macrophages; M2b macrophages showed the lowest expression of all three markers.

We next employed blocking antibodies against each integrin, as well as that against their collective β2-integrin binding partner, CD18, and tracked M2a macrophage migration and carcinoma aggregate dispersion by time-lapse imaging. We also assessed the involvement of ICAM-1, the ligand for these integrins, on A549 cancer cells using a neutralizing antibody. We found that specifically blocking CD11a, CD11b or CD18 on M2a macrophages or ICAM-1 on A549 aggregates significantly inhibited carcinoma aggregate dissociation, whereas blocking CD11c had no effect (Figure 5B). Intriguingly, blocking CD11b or CD18 did not affect the migration speed of M2a macrophages (Figure 5D). Supplementary Video S2 demonstrates how blocking either CD11b or CD18 can prevent aggregate dissociation without altering macrophage motility. Thus, we show that M2a macrophages migrate toward the aggregates and promote aggregate dispersion through a contact-dependent mechanism (Figure 5C) that employs the interaction of integrin and ICAM-1.

### M2a macrophages promote cancer cell migration in a transwell invasion assay

**Figure 6:**
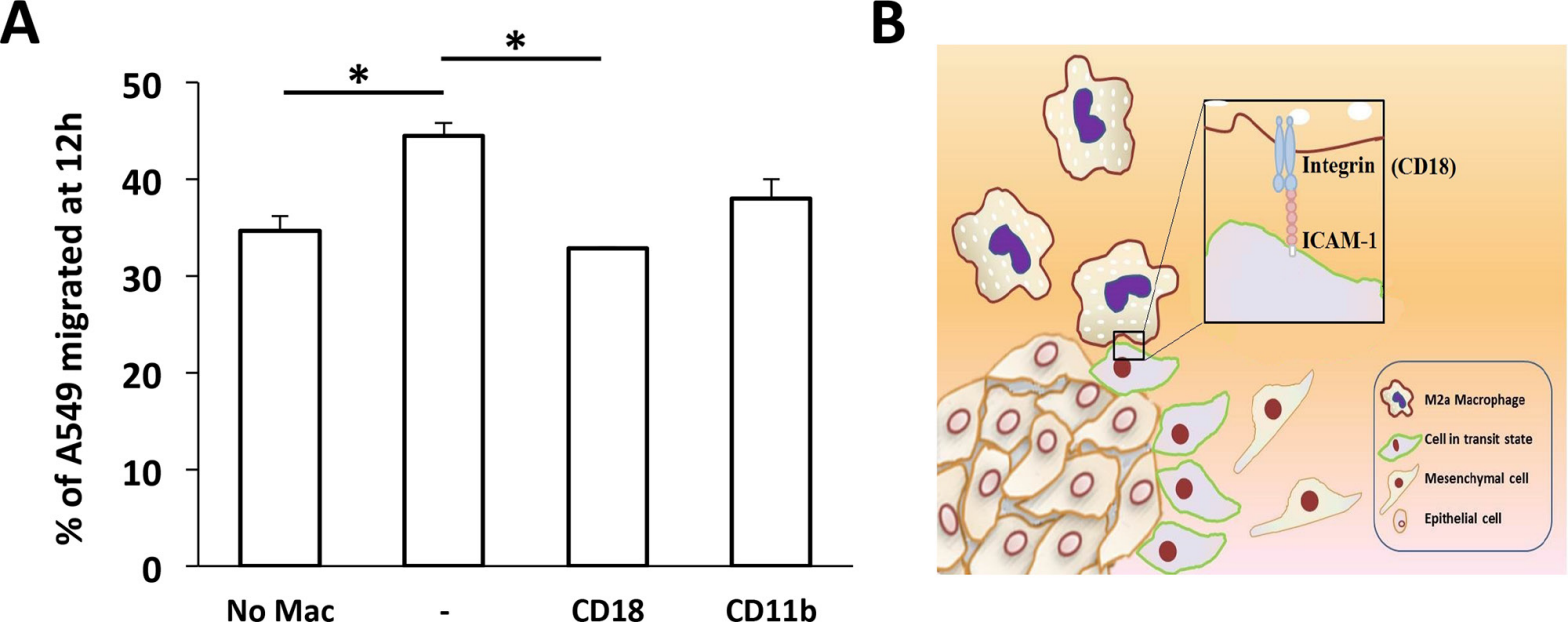
Transwell migration assay of A549 carcinoma cells **A.** Migration of A549 cells in the absence (No Mac) or presence of M2a macrophages without (−) or with the indicated blocking antibodies, measured after 12 h. Data are the mean ± SEM. *P < 0.05. **B.** Diagram showing the possible adhesion mechanism governing M2a-induced A549 aggregate dispersion via the interaction of ICAM-1 and β_2_ integrin.

To validate the role of this integrin-mediated interaction of M2a macrophages with tumor cells, HUVECs were grown as monolayers on collagen-coated wells, recapitulating the three-cell type culture in the microfluidic device. Blocking CD18 on M2a macrophages significantly reduced the proportion of migrating A549 cancer cells compared with that observed without the blocking antibody. We also found a reduced number of A549 cells that migrated in the presence of anti-CD11b blocking antibody; however, this reduction was not statistically significant (Figure 6A).

## DISCUSSION

Tumors are characterized by a highly heterogeneous local microenvironment comprising various immune cells that affect cancer cell dissemination. TAM function varies depending on the tumor type and extent of tumor progression, and recent studies have emphasized a role for these macrophages in promoting tumor dissemination [18, 27, 28]. Here, we investigated the influence of different macrophage subtypes on the EMT phenotype of lung carcinoma cells. We developed and demonstrated the capabilities of a 3D microfluidic assay to characterize the distinct roles played by different subtypes of macrophages on carcinoma aggregate dispersion, which is quantified as a metric of EMT. The three-cell culture system incorporated macrophages, lung adenocarcinoma A549 cells and HUVECs to partially mimic the *in vivo* microenvironment. Because macrophages can take on various phenotypes depending on the microenvironment, we differentiated human primary monocytes into different macrophage subtypes *in vitro*, namely M0, M1, M2a, M2b and M2c. Making use of our microfluidic system, we were able to position macrophages either in direct contact with or separated from carcinoma aggregates to establish their influence on the carcinoma cells.

Among the macrophage subtypes investigated, M1 and M2b exhibited the strongest ability to induce EMT, regardless of whether they were in contact or in separate compartments (Figure 3A). Previous studies have demonstrated a strong link between inflammatory cytokines and EMT induction [29–32]. M1 macrophages are distinguished from other macrophage subtypes by their ability to secrete significant amounts of pro-inflammatory cytokines, including IL-1β, IL-6 and TNFα [33]. M2 macrophages, in general, are characterized by their low production of pro-inflammatory cytokines, low levels of IL-12 and IL-23, and a high expression of IL-10. M2b, however, is an exception, as these cells retain high levels of pro-inflammatory cytokines concomitant with high levels of IL-10 and CD86 but low levels of IL-12 and arginase-1 [34]. Thus, the most extensive carcinoma aggregate dispersion induced by M1 and M2b macrophage subtypes might be attributed to the pro-inflammatory cytokines that they secrete, which requires further investigation. Similar results were also observed when HUVECs were excluded from the 3D culture, suggesting that endothelial cells do not alter the response of these subtypes of macrophages, as M1 and M2b are still dominant in triggering dispersion (Supplementary Figures S3 and S4).

Most interestingly, M2a macrophages promoted significantly greater aggregate dispersion in “contact” rather than “separated” conditions (Figure 3B). We showed that M2a macrophages exhibited the fastest locomotion, especially when in close proximity to carcinoma aggregates, and they preferentially migrated towards the aggregates. This indicated that they may be responding to some soluble factors secreted by the carcinoma aggregates (Figure 4A and 4B). Numerous cancers express tumor-derived factors, such as CSF-1 and CCL2, to recruit monocytes and promote their differentiation to macrophages in the tumor microenvironment [5, 25, 35–37]. For lung cancer, high intra-tumoral concentrations of CXCR2 ligands (CXCL1, CXCL5, and CXCL8) and type 2 cytokines IL-4, IL-5, IL-10, and IL-13 have been reported for non-small cell lung cancer [38, 39]. In our study, A549 aggregates may have been secreting one or more of these factors to specifically attract M2a macrophages since this is the macrophage subtype that expresses the CXCR2 receptor [40, 41].

ICAM-1 is a surface glycoprotein associated with numerous inflammatory and immune responses, mediating intercellular adhesion through binding to the β2 integrins: CD11a/CD18, CD11b/CD18 and CD11c/CD18 [42–44]. Specifically, ICAM-1-CD11a/CD18 interaction promotes the transendothelial migration of leukocytes from the capillary bed to the surrounding tissues. ICAM-1 also promotes the migration of other cell types, including cancer cells [45–47]. Using the 3D microfluidic assay, we showed that ICAM-1 was involved in carcinoma aggregate cell dissociation and migration (Figure 5B). Others have also shown a role for ICAM-1 during tumor metastasis, particularly invasion and migration [47–49]. ICAM-1 could only be detected in carcinoma cells from a few patients with gastric cancer, whereas all of the metastatic carcinoma cells from peritoneal effusions exhibited high ICAM-1 expression. ICAM-1 also correlated significantly with the proportion of tumor-infiltrating leukocytes, including macrophages [46]. Among oral cancers, ICAM-1 is expressed predominantly at the invasive front of tongue squamous cell carcinoma (SCC) and positively correlated with invasion, lymph node metastasis and increased density of circulating cancer cells in the blood and lymphatic vessels [50]. Furthermore, ICAM-1 expression correlated with increased macrophage infiltration within the SCC tumor [50]. However, in these studies, the phenotypes of the macrophages that infiltrated the tumors were not well characterized. Usami et al. showed that the macrophages were positive for CD163 expression and proposed that they were M2 macrophages in general [50]. They further showed that CD11b on the macrophages was involved in the interaction with ICAM-1 on squamous carcinoma cells and promoted their adhesion.

In our study, we showed that blocking CD11b on M2a macrophages—but not other macrophage subtypes—inhibited aggregate dispersion (Figure 5B), suggesting that the interaction between macrophages and carcinoma cells via ICAM-1/β2 integrins is necessary for their dissociation from the aggregate, an indicator of EMT. Figure 6B proposes a mechanism for this dispersal and suggests that M2a macrophages are attracted by and directly interact with the carcinoma aggregate to down-regulate junctional complexes. This process requires the binding of CD11b/CD18 on M2a macrophages to ICAM-1 on A549 lung carcinoma cells. To our knowledge, this is the first report to show that the M2a macrophage subtype specifically promotes the dissociation carcinoma cells through a CD11b and ICAM-1 interaction. Our study thus emphasizes the unique role of the M2a macrophage subtype for tumor cell dispersion through direct cell-cell contact.

Recruitment of the M2 macrophage subtype into the tumor microenvironment has been observed in numerous cancers [41, 51–54]. A similar ability of M2 macrophages to promote cancer invasion and dissemination has also been reported in mouse models of breast and lung cancer [51, 52]. TAMs that have infiltrated non-small lung cancer promote EMT intra-tumorally in addition to inducing EMT at the invasive front [6]. In the tissue array of a cohort of 491 patients, a positive correlation was observed between intra-tumoral macrophage prevalence, EMT marker expression, and tumor grade. In addition, the authors showed macrophage-derived TGFβ as the main inducer of the EMT phenotype [6]. Among the various macrophage subtypes, M2a and M2c could produce high levels of TGFβ. Although it is currently unclear how the interaction of M2a macrophages with the carcinoma aggregates via integrin/ICAM-1 can promote aggregate dissociation, it is likely that this interaction may act in synergy with secreted factors such as TGFβ to enhance EMT.

In summary, we describe a microfluidic-based approach that identifies a possible mechanism for carcinoma-macrophage signaling in EMT in 3D cultures. The distinct role of each subtype of macrophage was analyzed, with M1 and M2b subtypes showing the greatest ability to induce dispersion, whereas M2a acts predominantly through a contact-dependent mechanism. Tumor-infiltrating macrophages are either a mixture of all subtypes or they constitute a spectrum of the phenotypes while still retaining partial functions of each subtype. The proportion of each subtype may vary from one cancer type to another, promoting different levels of dissemination. Different populations of hematopoietic cells in the tumor microenvironment may therefore be exploited to design immunotherapeutic approaches and anti-metastatic drug screening.

## MATERIALS AND METHODS

### Generation of A549-H2B-mCherry stable cells and cancer aggregate formation

Human lung adenocarcinoma cells (A549) (#CCL-185; ATCC, Manassas, VA) were authenticated by the Centre for Translational Research and Diagnostics, National University of Singapore (November, 2014). A threshold of 80% was used to indicate authenticity, according to the standard ANSI/ATCC ASN-0002-2011. The authentication analysis was carried out using a Geneprint 10 system kit (Promega, Madison, WI) with capillary electrophoresis on the ABI 3130xl Genetic Analyzer (Life Technologies, Carlsbad, CA). STR marker calling was performed using Gene mapper V4.0 software (Life Technologies) and Deutsche Sammlung von Mikroorganismen and Zellkulturen (DSMZ, www.dsmz.de; includes ATCC, DSMZ, Riken, JCRB) and the Korean Cell Line Bank (KCLB, www.cellbank.snu.ac.kr; for SNU) were assessed.

Cells were transfected with PH2B_mCherry_IRES_puro2 plasmid (plasmid 21045, Addgene, Cambridge, MA) using FuGENE 6 (Roche, Basel, Switzerland). Transfected cells were cultured in the presence of puromycin (BD Biosciences, Franklin Lakes, NJ) for 48 h and FACS sorted for H2B-mCherry expression [26]. A549 aggregates were generated as previously described [56]. Briefly, A549 cells were resuspended at 5 × 10^4^ cells/ml in DMEM (Life Technologies) supplemented with 10% Fetal Bovine Serum (FBS (Life Technologies)) and 1% PenStrep (Life Technologies) and seeded into 90-mm culture dishes (VWR, Radnor, PA) that had been previously laser-ablated to produce a 100 × 100 array of microwells, pre-coated with 0.2% pluronic (Pluronic F108, BASF, Ludwigshafen, Germany) in PBS. Aggregates were retrieved after four days and sieved sequentially through 100-μm- and 40-μm-diameter cell strainers to yield aggregates of 40–100 μm in diameter. These samples were then enriched by centrifugation.

### Differentiation and polarization of monocyte-derived macrophages

All blood samples and procedures were approved by the Domain Specific Review Board (DSRB), National Healthcare Group, Singapore (Reference code: 08-352E). Written informed consent was given according to the principles expressed in the Declaration of Helsinki. Peripheral blood mononuclear cells were isolated from healthy donors' buffy coats (National University Hospital Blood Donation Center, Singapore) by Ficoll-Paque density gradient centrifugation, and monocytes were positively selected using CD14 microbeads (Miltenyi Biotec, Auburn, CA). The purity, as determined by flow cytometry, and viability, as measured by trypan blue exclusion of monocytes, were 98.07% ± 1.7% and 98.77% ± 0.8%, respectively.

Monocytes were maintained in Petri dishes in IMDM (Life Technologies) supplemented with 5% human serum and 100 ng/mL recombinant human M-CSF (Immunotools, Friesoythe, Germany) for five days to generate macrophages (hereafter labelled as M0). Macrophages were then either left untreated (M0) or polarized to M1 (20 ng/ml IFNγ(Roche) + 100 ng/ml LPS (Sigma-Aldrich, St. Louis, MO)), M2a (20 ng/ml IL-4 (Miltenyi Biotec)), M2b (human IgG (Jackson Immunoresearch Lab, West Grove, PA) coated wells + 100 ng/ml LPS (Sigma-Aldrich)) or M2c (20 ng/ml IL-10 (Miltenyi Biotec)) [24] for at least 24 h. Cell viability was assessed with a Live/Dead assay (Calcein/Ethidium Bromide (Life Technologies)).

### Fabrication of microfluidic device and generation of the tumor microenvironment

Polydimethyl siloxane (PDMS (Dow Corning, Midland, MI)) was selected for fabrication of the tissue culture microfluidic device (Figure 1A). A PDMS replica was made by soft lithography from patterned wafers (SU-8). Coverslips were plasma bonded to the PDMS micropattern to create closed chambers. The device consisted of two inner adjacent channels (‘2’ and ‘3’; Figure 1B) and two outer media channels (‘1’ and ‘4’; Figure 1B).

HUVECs (Lonza, Basel, Switzerland) were cultured in EGM-2 endothelial cell growth media (Lonza) and grown as a confluent monolayer in channel ‘4’.

To create a “contact condition”, a type I collagen gel solution (2.5 mg/ml, pH 7.4) was introduced into channels ‘2’ and ‘3’. Approximately 30–40 tumor spheroids plus 2500–3000 macrophages in the collagen gel solution were introduced into channel 2 and allowed to polymerize via thermal cross-linking. The average distance between the HUVEC monolayer and carcinoma spheroids was 200 μm, which facilitated rapid cell-cell communication. DMEM was subsequently introduced to the other empty media channel and changed on a 24-h cycle. Cells within the collagen compartment were imaged by confocal microscopy (FluoView 1000, Olympus, Tokyo, Japan).

For the “separated condition”, 2500–3000 macrophages were mixed with the collagen gel solution and introduced into channel ‘3’, while 30–40 A549 carcinoma aggregates in collagen gel were injected into channel ‘2’. All experiments were conducted in the presence of HUVECs unless otherwise stated.

### Immunocytochemistry

To characterize the M1 and M2a macrophage phenotypes in the device, cells were stained with the specific surface markers CD80 (clone 2D10.4) (eBioscience, San Diego, CA) and CD209 (clone B-2) (Santa Cruz, Dallas, TX), respectively. To assess the EMT of A549 aggregates, they were stained with the epithelial marker E-cadherin (Life technologies). Briefly, cell culture medium was removed from the microfluidic device, and the channel(s) containing the cells were rinsed in cold PBS and then fixed in 4% paraformaldehyde (PFA) (Sigma-Aldrich) for 15 min at room temperature. Then, 0.1% Triton-X (Sigma-Aldrich) was added for 10 min before blocking for 2 h at room temperature with PBS containing 5% BSA. After blocking, the samples were stained with CD80 (1:100), CD209 (1:50) or E-cadherin (1:100). The secondary antibody used was Alexa Fluor 488-conjugated anti-mouse IgG antibody (Life Technologies). Fluorescent images were obtained by confocal microscopy.

### Image processing and analysis

Three-dimensional image stacks (100 μm range) of each tumor aggregate were acquired by confocal microscopy with a 20× objective lens (N.A. = 0.4). Images were acquired at 0 h and 36 h. Aggregate normalized dispersion was quantified using Imaris 6.0 software (Bitplane, Zurich, Switzerland) following a previously reported method [26]. For clarity, a detailed description can be found in the Supplementary Material.

For live-cell imaging, macrophages were stained with 5 μM green cell tracker CMFDA (Life Technologies). Macrophage migration was tracked by capturing confocal image stacks (4-μm slice thickness) every 10 min for up to 12 h with a 10 × (N.A. = 0.3) objective lens. Time-lapse confocal z-stacks of A549 aggregates and macrophages were acquired simultaneously to generate video clips (See Supplementary videos). The macrophage mean migration speed was calculated by Imaris from the recorded trajectory of an individual nucleus (Supplementary Material). The mean migration speeds were then averaged for all macrophages. Methods for calculating the macrophage radial velocity can be found in Supplementary Material and Supplementary Figure S1.

### Flow cytometry

Polarized macrophages were washed once with PBS containing 2 mM EDTA and further incubated in PBS-EDTA at 37°C for 5 min. Cells were detached using a scraper and resuspended in FACS buffer (PBS containing 5% (vol/vol) human serum) and then stained with fluorochrome-conjugated antibodies for 15 min at room temperature. Labelled cells were washed once with FACS buffer and measured using an LSR Fortessa flow cytometer (BD Biosciences). Cells were also stained with isotype-matched antibodies to determine background staining. All data were analyzed using FlowJo software (Tree Star Inc., Ashland, OR).

### Blocking experiments

Blocking antibodies (10 μg/ml) against CD11a (Thermo Fisher Scientific, Waltham, MA), CD11b (eBioscience, San Diego, CA), CD11c (R&D Systems, Minneapolis, MN), and CD18 (R&D Systems) were used on M2a macrophages, and an ICAM-1 blocking antibody (R&D Systems) was used on A549 aggregates. Cells were incubated with respective blocking antibodies for 1 h prior to mixing with the collagen gel solution and then injected into the microfluidic device. The blocking antibody was also added to culture media in the microfluidic lateral channels. Cells were cultured in DMEM supplemented with 10% FBS and 1% PenStrep that was inactivated by heating at 56°C for 45 min (decomplemented). Aggregate dispersion was evaluated at 0 h and 12 h and macrophage speed at 0 h and 6 h.

### Transwell migration assay

Transwell plates (24-well, 8-μm pore size; Sigma-Aldrich) were used to conduct the migration assay. The lower chambers of the plate were collagen-coated and filled with EGM-2 medium containing HUVECs. After 4 h, the medium in the lower chamber was replaced with fresh media. A549 cells and M2a macrophages either without blocking or pre-blocked with CD18 or CD11b were added to the upper chamber in decomplemented DMEM. The plate was incubated at 37°C with 5% CO_2_, and the cells were allowed to migrate for 12 h. Cells on the upper surface of the filters were removed using cotton swabs, whereas cells that migrated to the lower surface were fixed and imaged by confocal microscopy with a 10× objective lens (N.A. = 0.3). The percentage of migrated A549 cells with respect to the total number of seeded cells was calculated considering a total of 128 regions of interest.

### Statistical analysis

All data are expressed as the mean ± S.E.M. An unpaired Student's *t*-test was used to determine significance. Measurements were calculated by averaging the mean values of at least three microfluidic devices, and each device represents one independent experiment. For integrin surface expression on macrophage subtypes, a one-way ANOVA with Tukey post-test was used to calculate significance.

## SUPPLEMENTARY MATERIALS FIGURES AND VIDEOS


