# Supplementary material for: Contact-dependent carcinoma aggregate dispersion by M2a macrophages via ICAM-1 and β2 integrin interactions
Source: Oncotarget. 2015 Jul 30;6(28):25295–307. doi: 10.18632/oncotarget.4716 (PMC4694832; doi:10.18632/oncotarget.4716)
Supplement: Supplementary file 1 [file oncotarget-06-25295-s001.pdf]

SUPPLEMENTARY MATERIALS

Calculation of normalized aggregate dispersion

The spatial coordinates of the centroids of all nuclei were determined by IMARIS. If there were N nuclei in a given spheroid, the geometric centers of nuclei were represented by:

$$(x_i,\ y_i,\ z_i),\ i = 1,\ 2,\ ...N$$

The aggregate center was then represented as:

$$\bar{x} = (\sum_i x_i)/N$$

$$\bar{y} = (\sum_i y_i)/N$$

$$\bar{z} = (\sum_i z_i)/N$$

Thus, the standard deviation of nuclei was calculated as:

$$\sigma_x^2 = \sum_i (x_i - \bar{x})^2/N$$

$$\sigma_y^2 = \sum_i (y_i - \bar{y})^2/N$$

$$\sigma_z^2 = \sum_i (z_i - \bar{z})^2/N$$

The dispersion was: $\Delta = \sqrt{\sigma_x^2 + \sigma_y^2 + \sigma_z^2}$.

Finally, the dispersion was normalized (Δ/Δ_0), where the normalizing value (Δ_0) was the dispersion value at $t = 0$.

Computation of macrophage migration speed and radial velocity

For a given macrophage, the migration speed is automatically computed by IMARIS, as follows. Spatial coordinates of the centroid at each time step, t, are represented as: (x_t, y_t, z_t) with $t = 0, 1, 2, ..N$. The macrophage displacement at each t is measured as:

$$D = \sqrt{D_x(t,\ t-1)^2 + D_y(t,\ t-1)^2 + D_z(t,\ t-1)^2}$$
$$D_x(t,\ t-1) = x_t - x_{t-1}$$
$$D_y(t,\ t-1) = y_t - y_{t-1}$$
$$D_z(t,\ t-1) = z_t - z_{t-1}$$

The total path length is defined as:

$$L = \sum_{t=t_0+1}^{t_N} \sqrt{D_x(t,\ t-1)^2 + D_y(t,\ t-1)^2 + D_z(t,\ t-1)^2}$$

and the mean migration speed S_M of each macrophage is:

$$S_M = \frac{L}{t_N - t_0}.$$

For the radial velocity, instead, assuming the center of the A549 aggregate is at the origin (0, 0, 0), the distances from the origin at time t_0 and t_N (r_0 and r_N) are calculated:

$$r_0 = \sqrt{x_0^2 + y_0^2 + z_0^2} \quad r_N = \sqrt{x_N^2 + y_N^2 + z_N^2}$$

The radial displacement between t_0 and t_N is:

$$r_N - r_0 = \sqrt{x_N^2 + y_N^2 + z_N^2} - \sqrt{x_0^2 + y_0^2 + z_0^2}$$

and the radial velocity of a given macrophage is, correspondingly:

$$v_r = dr/dt \cong (r_N - r_0)/(t_N - t_0).$$

SUPPLEMENTARY FIGURES AND VIDEOS

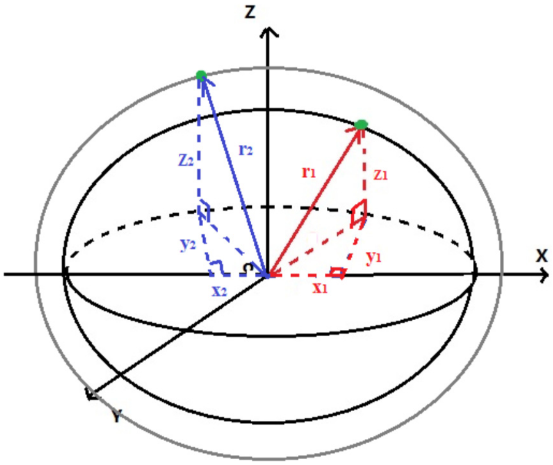

Supplementary Figure S1: A demonstration of the spatial coordinates of a macrophage at two different time points (Red: t_1; Blue: t_2).

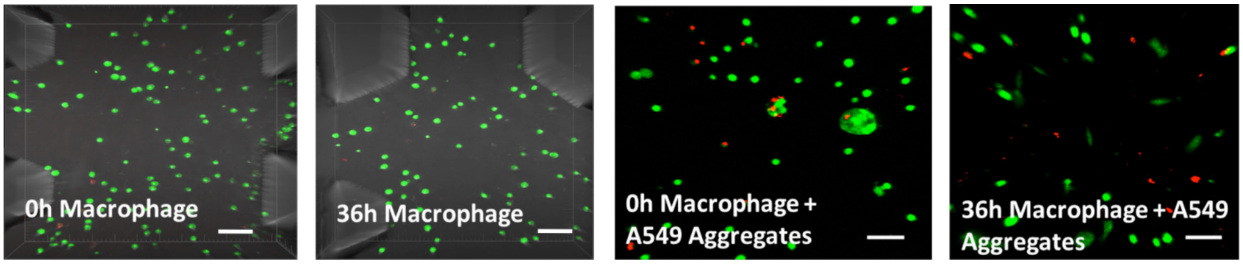

Supplementary Figure S2: Live/dead cell assay of M0 macrophages in the microfluidic device, either in mono-culture or co-culture conditions at 0 h and 36 h (green: live cells, red: dead cells). Scale bar, 50 µm.

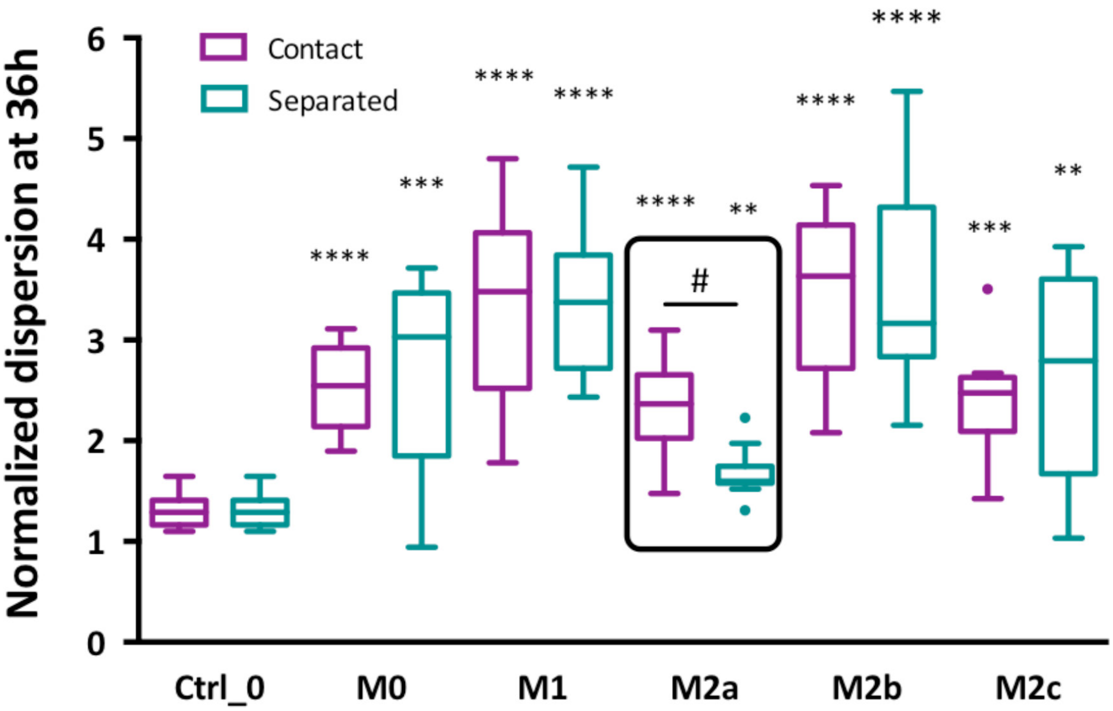

Supplementary Figure S3: Various subtypes of macrophages inducing cancer aggregate dispersion without co-culture with HUVECs. Data shown as box plot with Tukey outliers. Ctrl_0 represents the control without macrophages. Statistical calculations are compared to the no macrophage condition (i.e. Ctrl_0), where *$P < 0.05$, **$P < 0.01$, ***$P < 0.001$ and ****$P < 0.0001$. # indicates a statistical calculation between "contact" versus "separated" culture conditions, where #$P < 0.001$.

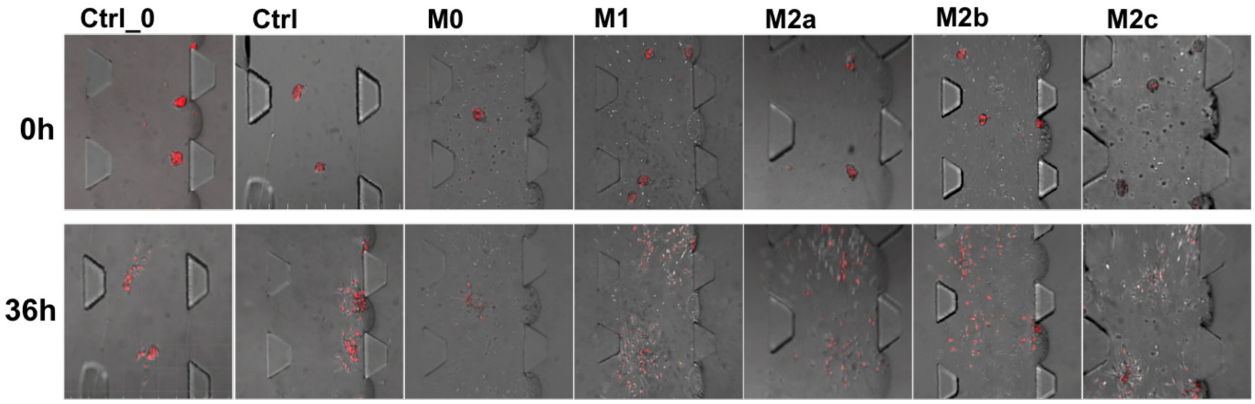

Supplementary Figure S4: Images of macrophage subtypes inducing A549 aggregate dispersion under "contact" conditions at 0 h or after culture for 36 h. Red: mCherry A549 nuclei. Ctrl_0 represents the control without HUVECs and without macrophages. Ctrl represents the control with HUVECs but without macrophages.

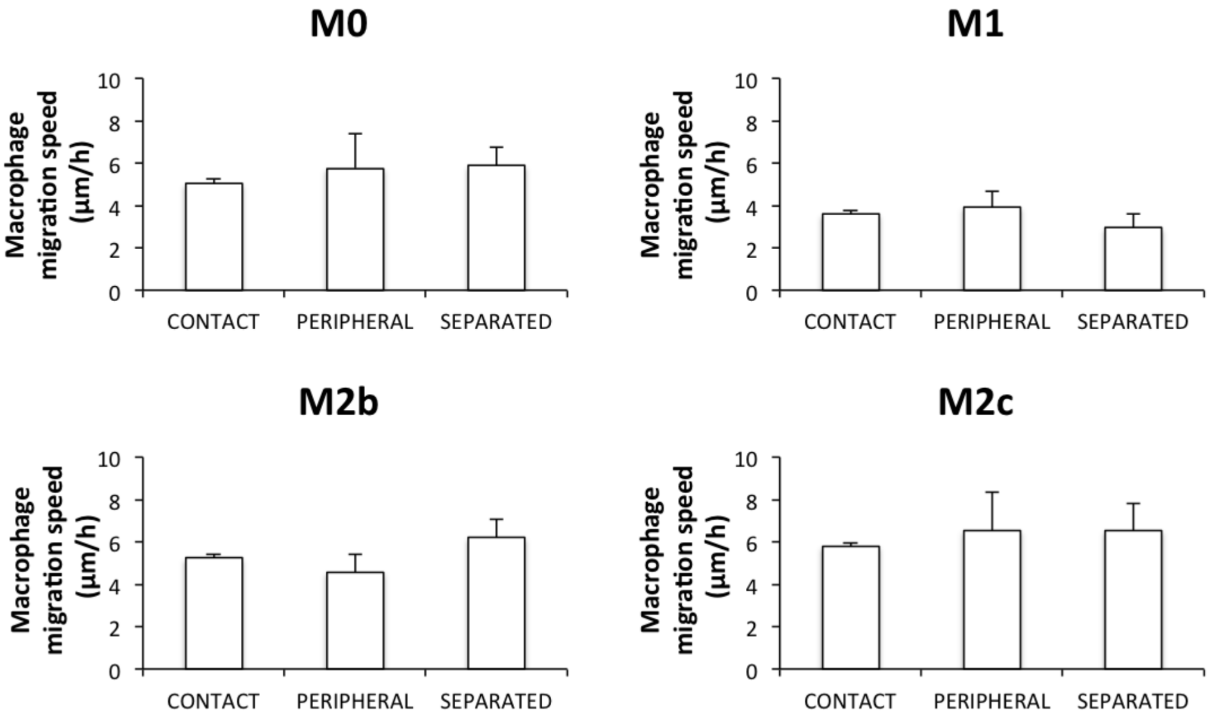

Supplementary Figure S5: Migration speed of M0, M1, M2b and M2c subtypes situated ≤50 µm (contact) or ≥50 µm (peripheral) from the carcinoma aggregates or grown under "separated" conditions.

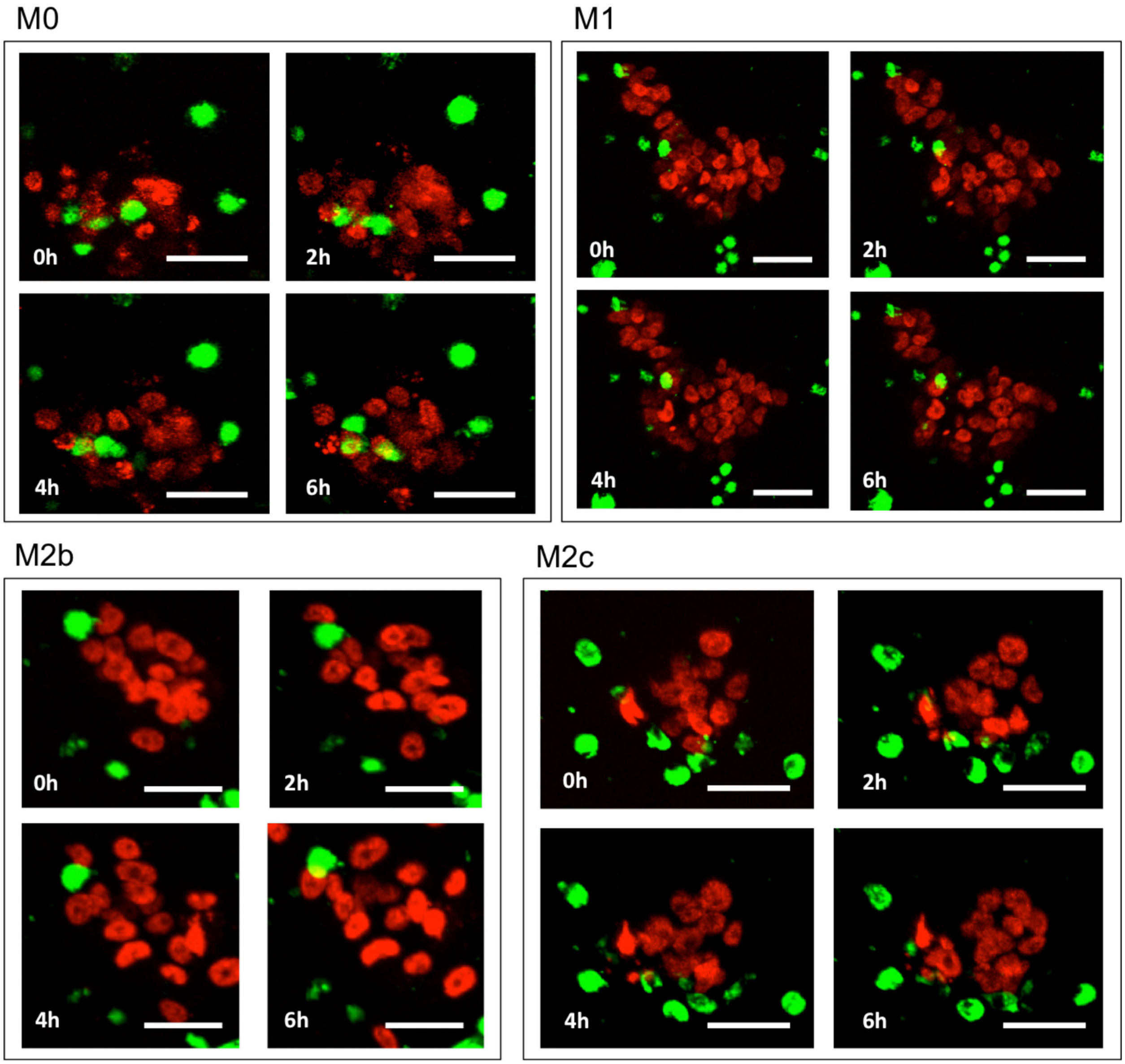

Supplementary Figure S6: Time-lapsed images of the M0, M1, M2b and M2c subtypes under "contact" conditions at 0 h, 2 h, 4 h, 6 h. Scale bars, 50 µm.

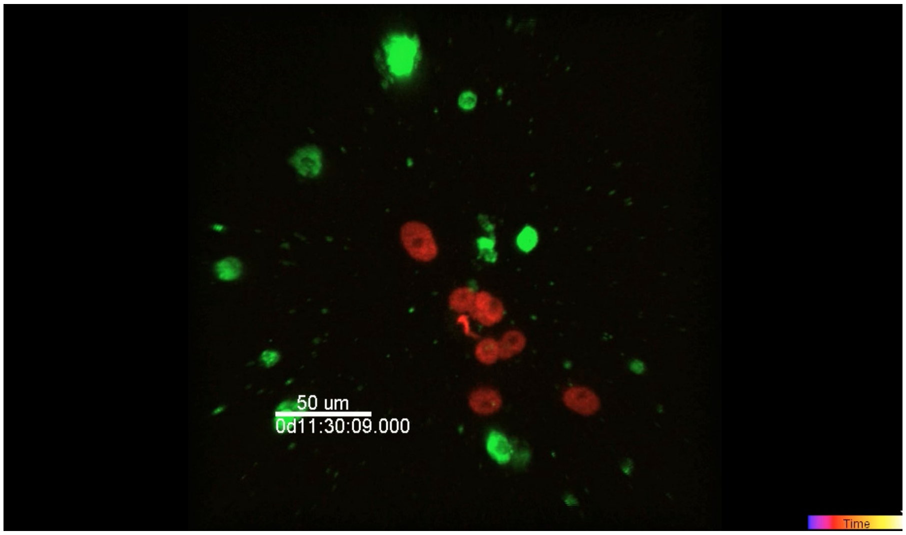
50 um
0d11:30:09.000
Time

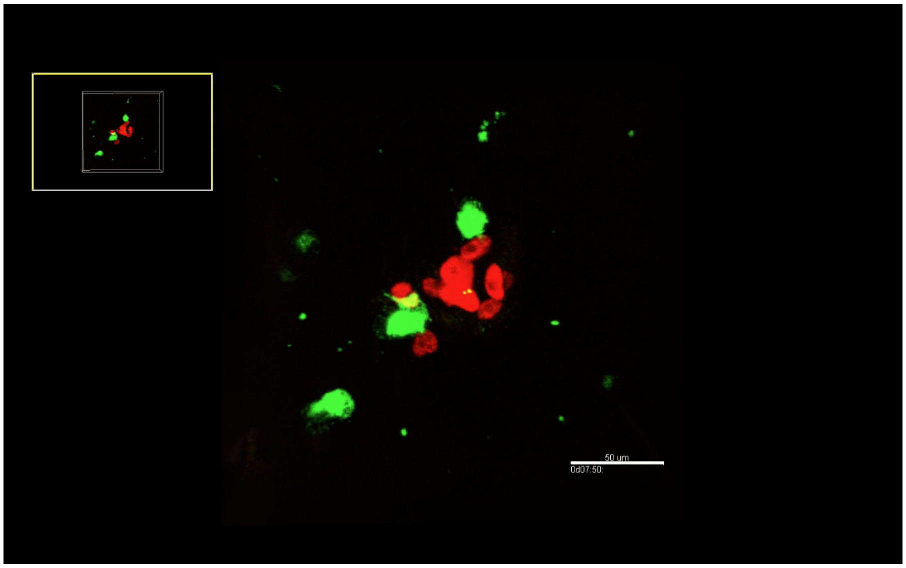

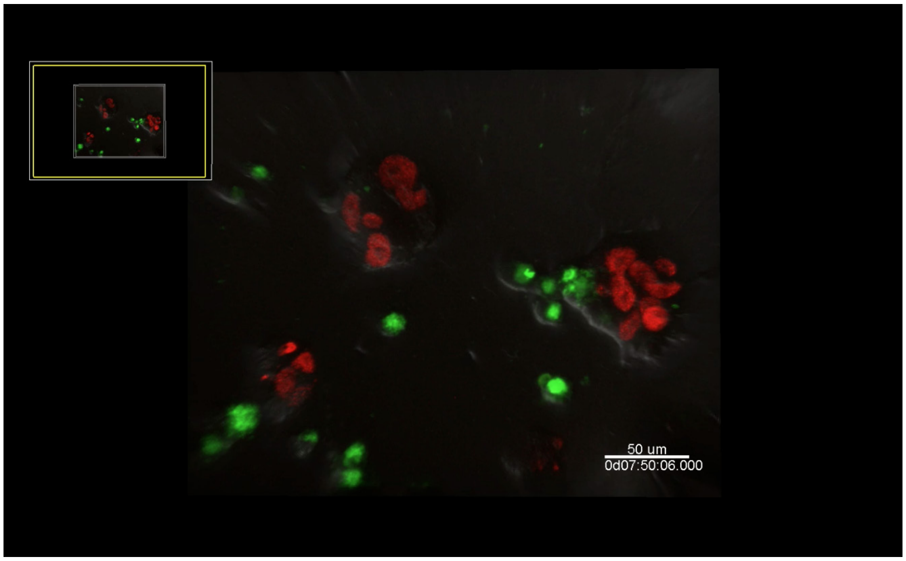
50 um
0d07:50:06.000

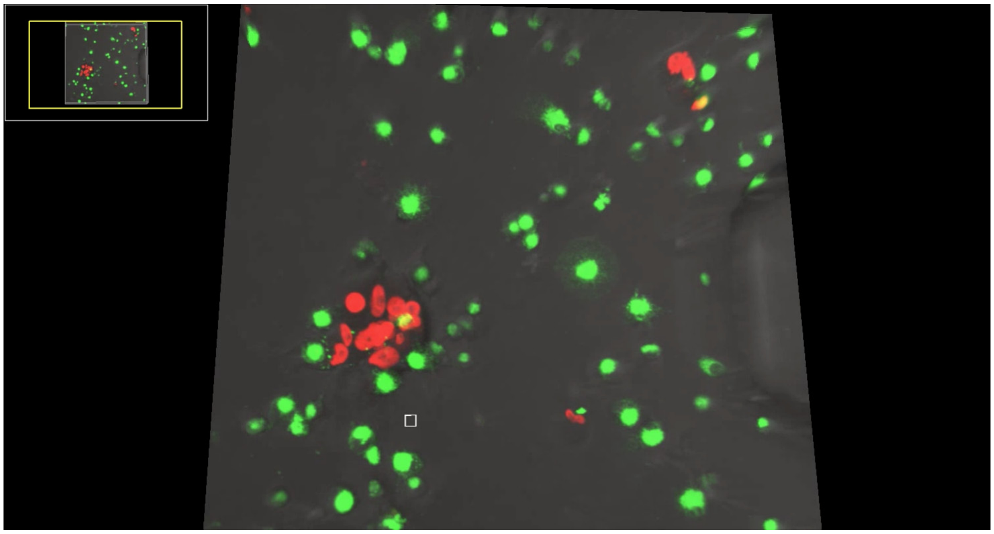

Supplementary Video S1: Time-lapsed analysis of various subtypes of macrophages on inducing A549 aggregate dispersion.

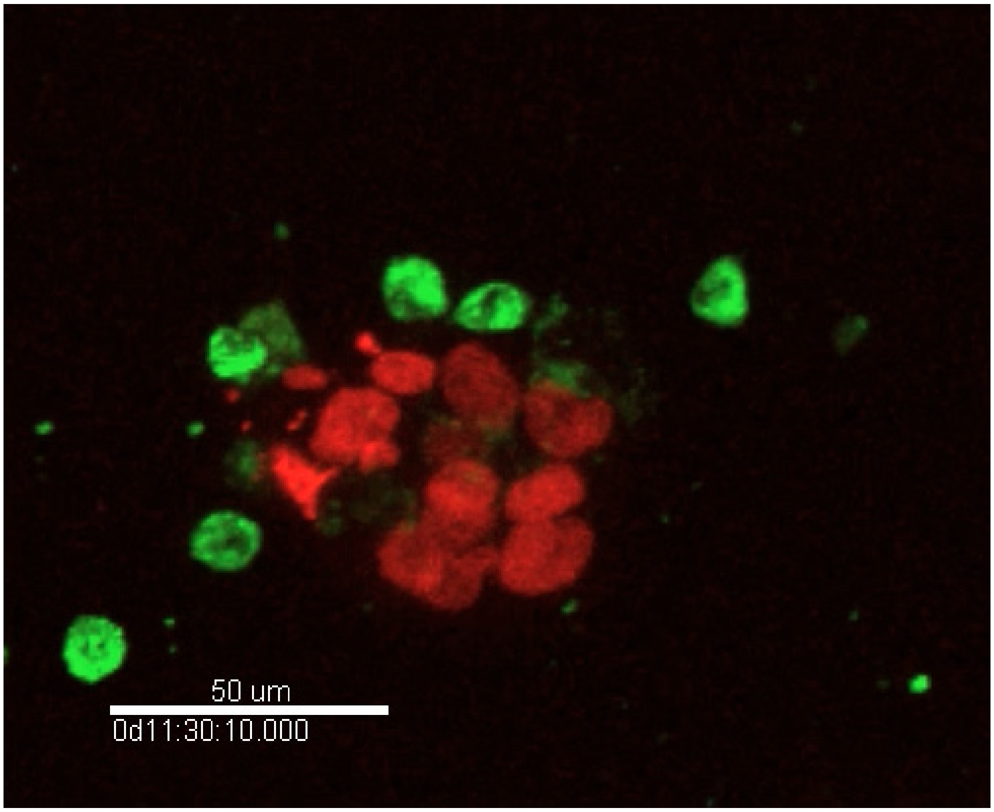

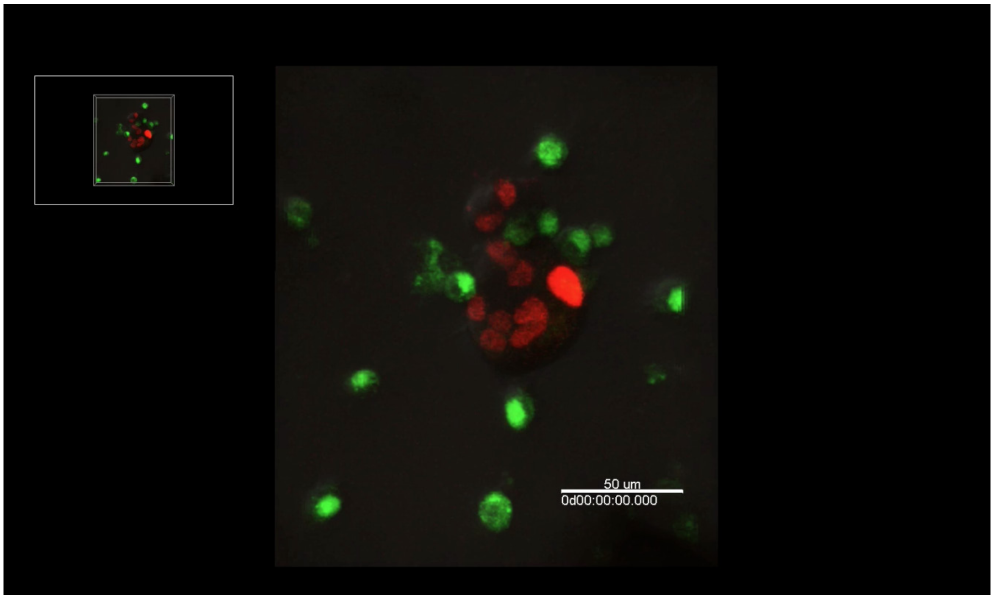
50 um
0d00:00:00.000

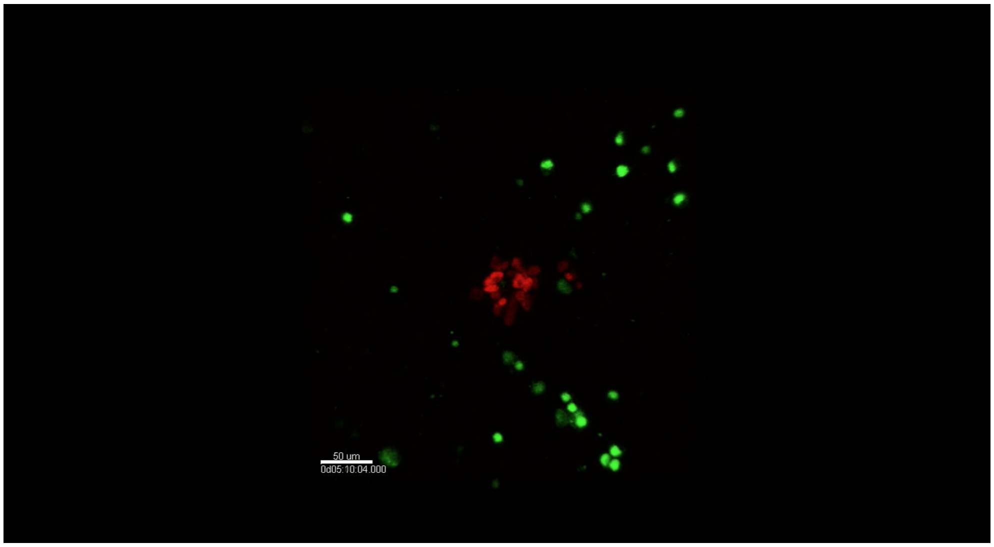
50 um
0d05:10:04.000

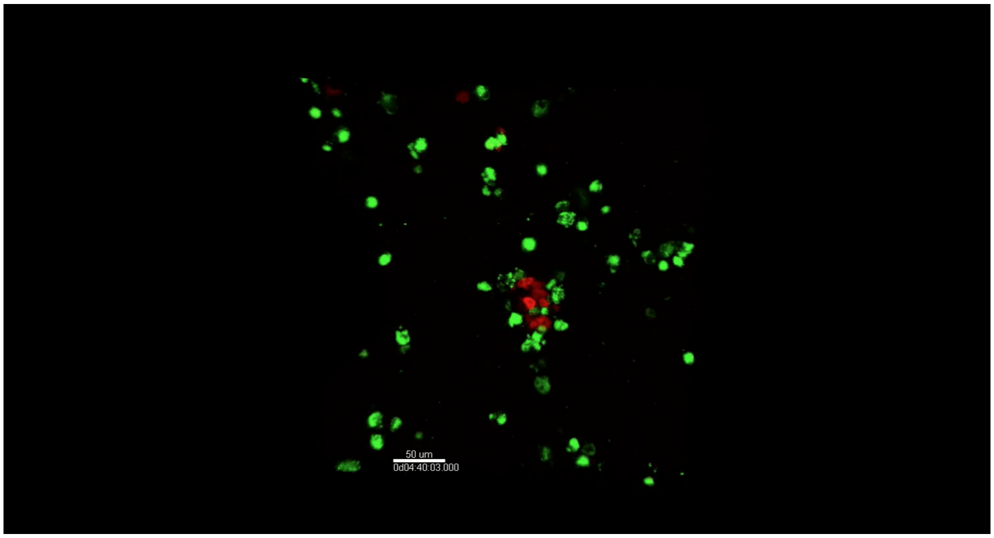
50 um
0d04:40:03.000

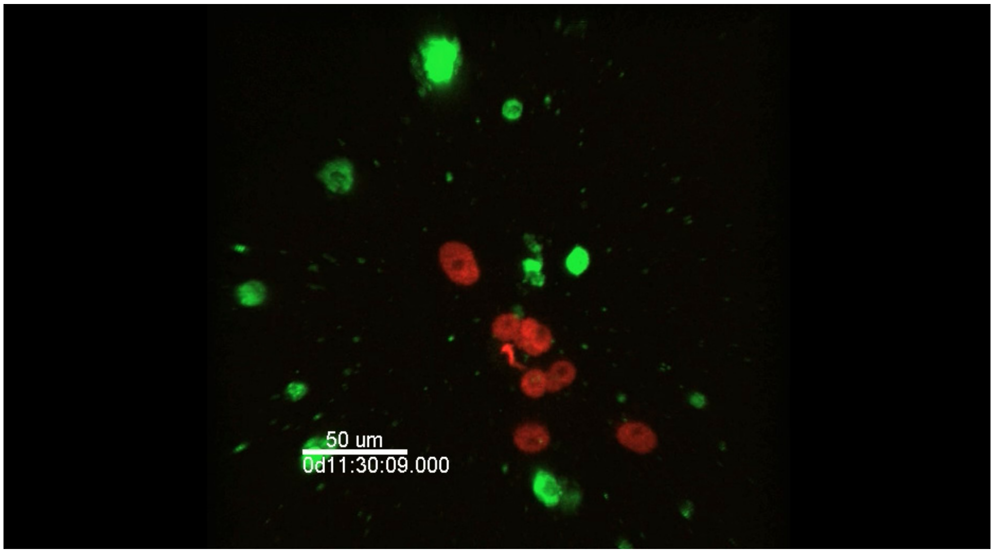
50 um
0d11:30:09.000

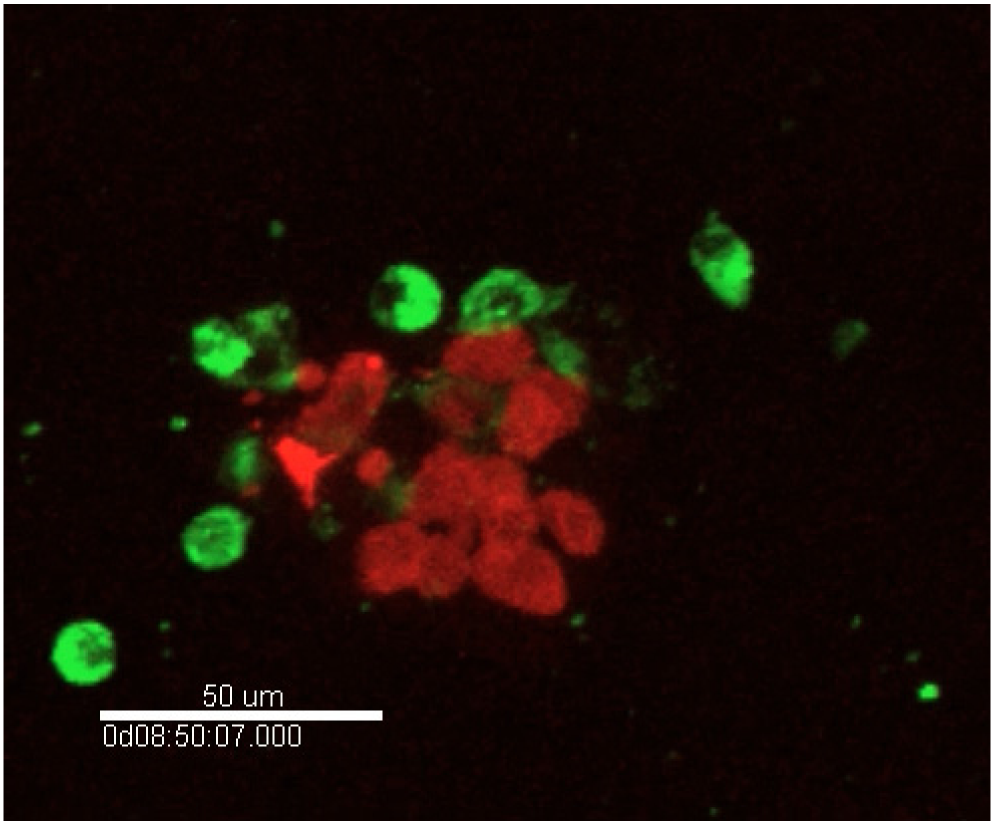

Supplementary Video S2: Time-lapsed analysis on M2a macrophages on inducing A549 aggregate dispersion in the presence of various blocking antibodies.
